# Supplementary figures and images for: The SMC5/6 complex prevents genotoxicity upon APOBEC3A-mediated replication stress
Source: EMBO J. 2024 Jun 17;43(15):3240–55. doi: 10.1038/s44318-024-00137-x (PMC11294446; doi:10.1038/s44318-024-00137-x)

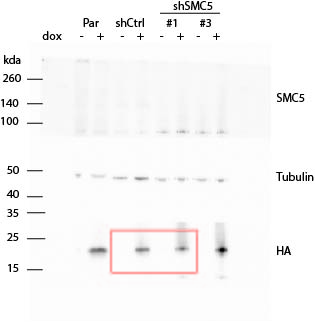

Supplement: Supplementary file 5 — Source data Fig. 1 [file 44318_2024_137_MOESM5_ESM.zip › Figure 1/1D/Fig 1D_HAexposure.jpg]

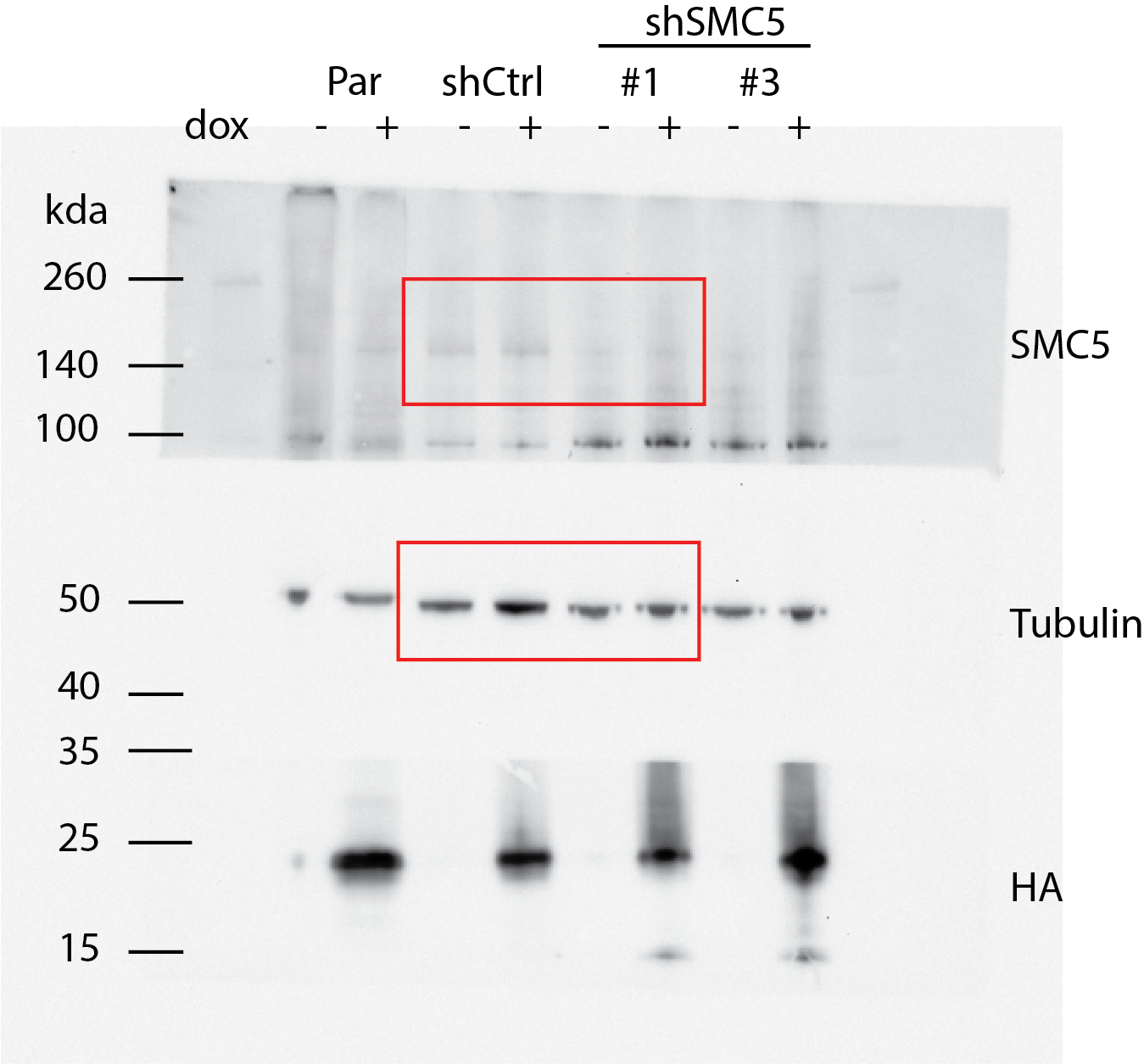

Supplement: Supplementary file 5 — Source data Fig. 1 [file 44318_2024_137_MOESM5_ESM.zip › Figure 1/1D/Fig 1D_SMC5exposure.png]

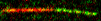

Supplement: Supplementary file 10 — Source data Fig. 6 [file 44318_2024_137_MOESM10_ESM.zip › Figure 6/6A/Panel 6a - Representative Fiber.tif]
