# Supplementary material for: The SMC5/6 complex prevents genotoxicity upon APOBEC3A-mediated replication stress
Source: EMBO J. 2024 Jun 17;43(15):3240–55. doi: 10.1038/s44318-024-00137-x (PMC11294446; doi:10.1038/s44318-024-00137-x)
Supplement: Supplementary file 7 — Source data Fig. 3 [file 44318_2024_137_MOESM7_ESM.zip › yeast A3A figure source data_Jiayi_0424.pdf]

Figure 3A source data

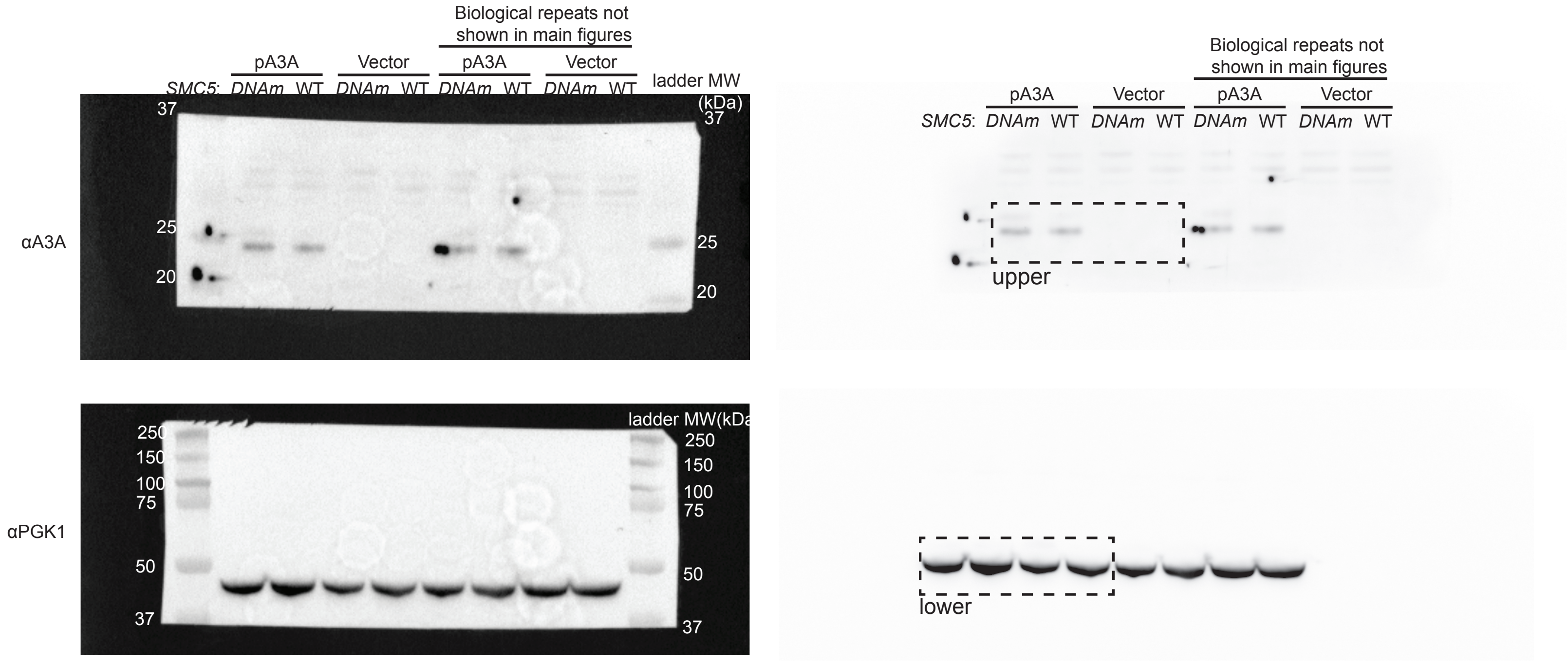

Figure 3B source data

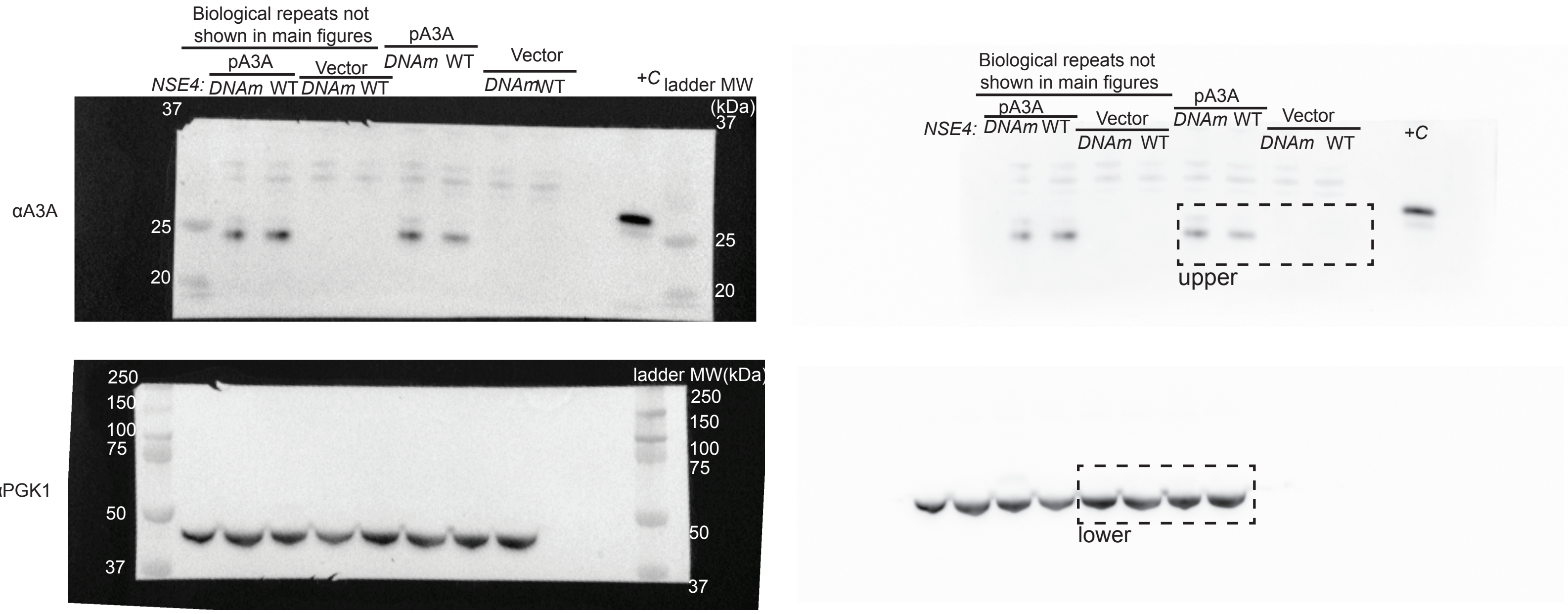

Different exposures than those of the same membranes shown to the right of each image, overlaid with the molecular weight markers channel

Exposures used in main figures

MW: molecular weight

Squares with dashed lines indicate cropped image parts used in the corresponding figure.
